# Population Pharmacokinetics and Exposure-Response Relationships of Naldemedine

**DOI:** 10.1007/s11095-018-2501-7

**Published:** 2018-10-02

**Authors:** Ryuji Kubota, Kazuya Fukumura, Toshihiro Wajima

**Affiliations:** Clinical Pharmacology & Pharmacokinetics, Project Management Department, Shionogi & Co., Ltd., 12F, Hankyu Terminal Bldg., 1-4, Shibata, 1-chome, Kita-ku, Osaka, 553-0012 Japan

**Keywords:** exposure-response, naldemedine, opioid-induced constipation, pharmacokinetic/pharmacodynamic analyses, population pharmacokinetics

## Abstract

**Purpose:**

To characterize population pharmacokinetic (PK) of naldemedine, to identify factors which influence naldemedine PK, and to evaluate their clinical relevancy based on exposure-response relationships.

**Methods:**

A population PK model was developed with pooled naldemedine concentrations from healthy subjects, patients with chronic non-cancer pain and opioid-induced constipation (OIC), and cancer patients with OIC. Exposure-response analyses were performed with efficacy (responder or non-responder) and safety (occurrence of gastrointestinal disorders or not) data in phase 2b and phase 3 studies.

**Results:**

Naldemedine plasma concentrations were adequately described by a 2-compartment model with first-order absorption and absorption lag time. The final model included the effects of age, creatinine clearance, race, and gender on apparent total clearance; the effects of body weight, health status, and food condition on apparent volume of central compartment; and the effect of age on first-order rate of absorption. When subjects took 0.2 mg of naldemedine once daily, the probability of spontaneous bowel movement (SBM) responders was predicted to be approximately 50%, while that of severe gastrointestinal disorders was predicted to be less than 3%. The influence of the covariates on PK was not considered clinically significant because similar efficacy and safety were expected based on the exposure-response analysis.

**Conclusions:**

The covariates are identified in the population PK analysis; however, no dose-adjustment is required for them based on the exposure-response analysis.

**Electronic supplementary material:**

The online version of this article (10.1007/s11095-018-2501-7) contains supplementary material, which is available to authorized users.

## INTRODUCTION

Naldemedine, an orally active peripherally-acting μ-opioid receptor antagonist (PAMORA), has been developed by Shionogi & Co., Ltd. (Shionogi) for treatment of opioid-induced constipation (OIC) in adult patients with pain who have been treated with opioids. Constipation is often associated with opioid treatment, primarily via activation of μ-opioid receptors in the gastrointestinal tract (1). Naldemedine acts to decrease the constipating effects of opioids by blocking opioid actions at peripheral μ-opioid receptors in the enteric nervous system (2–7). The drug was first approved in March 2017 in the United States for the treatment of OIC in adult patients with chronic non-cancer pain, including patients with chronic pain related to prior cancer or its treatment, who do not require frequent (e.g., weekly) opioid dosage escalation. This drug was subsequently approved in Japan for the treatment of opioid-induced constipation in adult patients with cancer and non-cancer pain (8,9). The recommended dosage for adults is 0.2 mg once daily, taken with or without food (10).

Systemic exposure to naldemedine was almost dose proportional within the tested dose range of 0.1 mg to 100 mg. In a multiple dose study, the area under the plasma concentration curve (AUC) on Day 1 and that at steady-state (AUC_ss_) on Day 10 were similar, suggesting that the pharmacokinetics (PK) of naldemedine are time-independent (10,11). Absorption of naldemedine in the fasted state was rapid (0.5 to 0.75 h), and slight accumulation at the maximum plasma concentration (C_max_) (1-fold to 1.3-fold) and AUC (1-fold to 1.2-fold) of naldemedine were observed. The relatively constant plasma trough concentration levels of naldemedine from Days 2 to 10 for all tested doses (3 mg, 10 mg, and 30 mg) in the multiple dose study suggest that the PK of naldemedine reached a steady state within 2 days of treatment. Following oral administration of 2 mg of [^14^C]-labeled naldemedine, the total amount of radioactivity excreted in the urine and feces was 57 and 35% of the administered dose of naldemedine, respectively (8). The amount of naldemedine excreted unchanged in the urine was approximately 16 to 18% of the administered dose. Benzamidine was the most predominant metabolite excreted in the urine and feces, representing approximately 32 and 20% of the administered dose of naldemedine, respectively.

In the dose-finding study in patients with chronic non-cancer pain and OIC (Study V9221), 0.1, 0.2, and 0.4 mg doses of naldemedine were evaluated (5). The proportion of SBM responders was significantly higher with naldemedine 0.2 mg (71.2%) and 0.4 mg (66.7%), but not with 0.1 mg (52.5%), *vs* placebo (39.3%) in the phase 2b study (Study No. 1107 V9221). Treatment-emergent adverse events were more common with naldemedine (0.1 mg: 66.1%; 0.2 mg: 67.2%; 0.4 mg: 78.6%) than placebo (51.8%); the most common treatment-emergent adverse event was diarrhea. In the pivotal Phase 3 studies, the proportion of responders in both trials was significantly higher with naldemedine than with placebo in Study 1314 V9231 (47.6% in the naldemedine group *vs* 34.6% in the placebo group) and in Study 1315 V9232 (52.5 *vs* 33.6%) in the phase 3 studies (2). Treatment-related adverse events were noted in 59 (22%) of 271 patients in the naldemedine group and 45 (17%) of 272 in the placebo group in Study 1314 V9231, and in 54 (20%) of 271 patients in the naldemedine group and 31 (11%) of 274 in the placebo group of in Study 1315 V9232.

The naldemedine PK had been characterized in healthy subjects; however, the phase 1 studies did not reflect physiological and demographic background of OIC patients. Inter-individual variability in naldemedine PK needs to be addressed with data including target patients. The purposes of this study were to identify intrinsic and extrinsic factors which influence naldemedine PK by developing a population PK model and to explore the exposure-response relationships of naldemedine based on the estimated PK parameters and efficacy and safety data.

## MATERIALS AND METHODS

### Data Used in the Population PK Analysis

A population PK model was developed with pooled naldemedine concentrations from healthy subjects, patients with chronic non-cancer pain and OIC, and cancer patients with OIC as described in Table I. Blood sampling times for PK analysis in each study are shown in Supplemental Table S1. All studies were performed in accordance with the Declaration of Helsinki, the International Conference on Harmonization (ICH) E6 Guidance for Good Clinical Practice and all other applicable regulatory requirements. Study protocols were approved by the responsible institutional review boards. Written informed consent for each clinical study was obtained from all subjects.

**Table I:** Summary of the Observed Concentrations included in the Population PK Analysis

Study title	Study No.	Dose (mg)	Number of subjects ^a^	Number of subjects with only BLQ records	Number of plasma concentrations ^b^	Number of BLQ records
Phase 1 studies						
Single dose study (healthy Japanese)	0824 V9211	0.1	6	0	108	6
		0.3	6	0	108	6
		1	6	0	108	6
		3	6	0	108	6
Multiple dose study (healthy Japanese)	0917 V9213	3	9	0	414	9
Mass balance study	1016 V9215	2	12	0	307	86
DDI study with cyclosporine (P-gp inhibitor)	1202 V9218	0.4	13	0	260	14
Thorough QTc study	1204 V9219	0.2	52	0	572	53
		1	49	0	539	49
BA/ FE study (to-be-marketed tablet)	1311V921A	0.2	18	0	972	86
DDI study with rifampin (CYP3A inducer)	1403V921D	0.2	14	0	280	20
Renal impairment study	1401V921B	0.2	38	0	919	51
Hepatic impairment study	1402V921C	0.2	24	0	480	27
DDI study with itraconazole/ fluconazole (CYP3A inhibitors) (healthy Japanese)	1502V921E	0.2	28	0	560	49
Phase 2 studies						
Phase 2 OBD POC study in patients with chronic non-cancer pain	1007 V9214	0.01	9	0	171	42
		0.03	9	0	171	36
		0.1	9	0	169	15
		0.3	9	0	171	11
		1	9	0	170	9
		3	9	0	171	9
Phase 2b dose finding study in patients with chronic non-cancer pain	1107 V9221	0.1	9	0	75	9
		0.2	9	0	76	9
		0.4	10	0	79	10
Phase 2b dose finding study in cancer patients	1108 V9222	0.1	10	0	59	1
		0.2	16	0	95	0
		0.4	12	0	71	0
Phase 3 studies						
Phase 3 DBT study #1 in patients with chronic non-cancer pain	1314 V9231	0.2	259	31	721	111
Phase 3 DBT study #2 in patients with chronic non-cancer pain	1315 V9232	0.2	261	44	723	155
Phase 3 DBT Study in Japanese Cancer Patients	1331 V9236	0.2	97	0	276	5
Phase 3 long-term safety study in Japanese patients with chronic non-cancer pain	1336 V9238	0.2	43	0	85	0
Phase 3 long-term safety study, in Japanese patients with chronic non-cancer pain receiving oxycodone therapy	1339 V9239	0.2	10	0	20	2

*BA* Bioavailability, *BLQ* Below the limit of quantification, *DBT* Double blind test, *DDI* Drug-Drug Interaction, *FE* Food effect, *OBD* Opioid-induced bowel dysfunction, *POC* Proof of concept

^a^ Subjects with only BLQ records were included

^b^ BLQ records were included

Background data available for subjects were summarized and used as candidate covariates: age, body weight, body mass index (BMI), albumin (ALBU), aspartate aminotransferase (AST) and alanine aminotransferase (ALT), total bilirubin (Tbil), and creatinine clearance (CLcr) at baseline as continuous data, and gender (male, female), race/ethnicity (“White” or “non-White”, “Japanese” or “non-Japanese”, “Hispanic or Latino” or “non-Hispanic or non-Latino”), health status (healthy subjects/ Patients with chronic non-cancer pain and OIC/ Cancer patients with OIC), dosing conditions (dosing in the fasted/fed state, with/without concomitant use of P-gp/CYP3A inhibitor/inducer), and formulation (“solution or suspension”, “phase 1 or 2 tablet”, “phase 3 tablet”) as categorical data. Background data at baseline were obtained from observations prior to or on the first day of dosing, or at screening if this value was not available. Categorical age (<65 years or ≥ 65 years) was also used. CLcr was calculated using the Cockcroft-Gault eq. (12). Data from the concomitant treatment period in the phase 1 DDI studies [1202 V9218, 1403V921D, and 1502V921E]), in which co-administrations of P-gp/CYP3A inhibitor/inducer (cyclosporine, rifampin, itraconazole, or fluconazole) with naldemedine were designed as the worst case scenario for each inhibitor/inducer (single dose of cyclosporine 600 mg, rifampin 600 mg for 17 days, itraconazole 200 mg for 7 days, fluconazole 200 mg for 7 days), were excluded from the population PK analysis because they were not clinically relevant to the OIC treatment. Food condition was assumed as fasted in phase 2b studies (1107 V9221 and 1108 V9222), in which the date/time of food intake had not been recorded. Food condition was defined as fasted in cases where naldemedine was administered more than 1 h after the food intake in phase 3 studies (1314 V9231, 1315 V9232, 1331 V9236, 1336 V9238, and 1339 V9239).

### Determination of Plasma Naldemedine Concentrations

The bioanalytical methods for determination of concentrations of naldemedine and its metabolites in human plasma were validated with the lower limit of quantification (LLOQ) being 0.01 ng/ml for naldemedine (11). Plasma samples were analyzed after solid phase extraction by a liquid chromatography/mass spectrometry/mass spectrometry (LC/MS/MS) method using the positive ion mode and multiple reaction monitoring. All the analytical methods were validated across the calibration range with respect to selectivity, recovery, accuracy, precision, and stability under a variety of conditions.

### Pharmacokinetic Analysis

The population PK analysis was performed by using the non-linear mixed effect modeling software NONMEM (version 7.3, ICON Development Solutions, US), with PREDPP library and NM-TRAN preprocessor (13). To support analyses by NONMEM, Perl-speaks-NONMEM (PsN) (version 4.2) and Xpose (version 4.2.1) were used (14). The first order conditional estimation with interaction (FOCE-I) method was used for the analysis.

The PK profiles of naldemedine were examined using conventional compartment disposition models with first order absorption based on the PK in healthy volunteers (11). To determine the basic structural PK model for naldemedine, 1-, 2- and 3-compartment models with first order absorption were tested. A population PK model was selected based on convergence of the estimation and covariance routines, the likelihood ratio test, PK parameter point estimates, their respective confidence intervals, and goodness-of-fit plots. Inter-individual variability was implemented using the exponential error structure that assumed log-normal distribution of individuals. Inter-individual variability for apparent total clearance (CL/F), apparent volume of central compartment (V/F) and first-order rate of absorption (Ka) were incorporated as the basic error structure. The exclusion of the inter-individual variability for apparent inter-compartmental clearance (Q/F) and apparent volume of peripheral compartment (Vp/F) was assessed in the model building process. Additive, proportional, and combination error structures were evaluated for the residual error.

After building a base model with selection of an error model for intra-individual variability, the influence of background data was assessed to build a covariate model. Age, body weight, BMI, gender, ALBU, AST, ALT, Tbil, CLcr, race, ethnicity, health status and dosing condition were tested as covariates of CL/F; age, body weight, BMI, gender, race, ethnicity, health status, and dosing condition were tested as covariates of Vc/F; and age, gender, health status, dosing condition, and formulation were tested as covariates of Ka. The covariates for Q/F and Vp/F were not evaluated since those models were unstable and could not be successfully estimated by NONMEM.

For continuous covariates, the power model shown in Eq. (1) was used.

1 $$ \mathrm{PKP}={\uptheta}_1\times {\left(\mathrm{COV}/\mathrm{median}\ \mathrm{of}\ \mathrm{COV}\right)}^{\uptheta 2} $$

where COV are values of the covariate and θ_1_, θ_2_ are typical values of model parameters to be estimated in the equation.

For binary and categorical covariates, a multiplicative model as shown in Eq. (2) was used.

2 $$ \mathrm{PKP}={\uptheta}_{\mathrm{CAT}=0}\times {\left({\uptheta}_{\mathrm{CAT}\_\mathrm{i}}\right)}^{\mathrm{CAT}\_\mathrm{i}} $$

where CAT_i is a series of indicator variables with a value of either 0 or 1 assigned (CAT_1, CAT_2, …, CAT_n representing the n levels of CAT; e.g., CAT_1 = 0 for male and CAT_1 = 1 for female), and θ_CAT = 0_ is the typical values of model parameters to be estimated when the individual categorical covariate index variable is equal to zero and θ_CAT_i_ is the i-th relative influence of model parameters to be estimated for categorical covariate index variable when CAT_i is equal to one. For race or ethnicity, “White or non-White”, “Japanese or non-Japanese”, “Hispanic or Latino, or non-Hispanic or non-Latino” were allowed to be tested in separate models in order to avoid confounding the estimation of covariate effects.

Covariate model was constructed by means of combination of forward selection after screening and stepwise backward deletion. After statistically significant covariates were chosen at the significance level of 0.05 based on the χ^2^ test (a decrease in the value of objective function value [OBJ] of less than −3.84 for one degree of freedom) in screening, they were assessed in order of magnitude of the decrease in OBJ to reduce NONMEM runs due to large numbers of covariates. The significance level of 0.05 based on the χ^2^ test was used for the forward selection as well. The significance level of 0.01 based on the χ^2^ test was used for the backward deletion (an increase in the value of OBJ of less than 6.64 for one degree of freedom).

The final model was evaluated by using the point estimates of PK parameters and their respective 95% confidence intervals. In addition, nonparametric bootstrapping with 200 replicates was applied to evaluate the distribution of the final parameter estimates by using PsN. The goodness-of-fit plots were generated for model diagnosis and prediction-corrected visual predictive checks (VPC) (15) with 1000 replicates of the data set were generated by using PsN.

The individual AUC_ss_ was calculated by using empirical Bayesian estimations based on the PK parameters in the final population PK model.

### Exposure-Response Analysis

Exposure-response analysis was used to evaluate the efficacy and safety data of the phase 2b and phase 3 studies (2,5). The PK/Efficacy analysis included the subjects of the population PK analysis or in the placebo group, who also had efficacy data for the exposure-response analysis obtained from phase 2b and phase 3 studies (1107 V9221, 1314 V9231, and 1315 V9232). The PK/Safety analysis included the subjects of the population PK analysis or in the placebo group, who also had safety data for exposure-response analysis obtained from phase 2b and phase 3 studies (1107 V9221, 1314 V9231, and 1315 V9232).

The following efficacy variables were used to evaluate the exposure-response relationship. In the phase 2b study (Study No. 1107 V9221), a spontaneous bowel movement (SBM) responder is defined as any subject whose frequency of SBMs per week within the last 2 weeks of the treatment period (28 days) was 3 times or more per week and had an average increase in the frequency of SBMs per week from a baseline of 1 or more. In the phase 3 studies (Study No. 1314 V9231 and 1315 V9232), an SBM responder was defined as having 9 positive response weeks or more out of the 12-weeks treatment period and 3 positive response weeks out of the last 4 weeks of the 12-weeks treatment period. A positive response week was defined as a week with both ≥3 SBMs and an increase of ≥1 SBM from the baseline. A subject with any insufficient response data was treated as a “non-responder”. The occurrence of treatment-emergent adverse events of gastrointestinal disorders as system organ class, which was the most frequently occurring type, was defined by the severity and assessed as safety variables.

No covariate was tested in the exposure-response analysis because no prognostic factors of the efficacy or safety were identified in the phase 2b and phase 3 studies (2,5). Efficacy and safety data in Study No. 1314 V9231 and 1315 V9232 were combined because these studies were conducted as pivotal phase 3 studies using the same study design and were of the same treatment duration. The individual AUC_ss_ values for naldemedine were estimated by empirical Bayes estimation based on the population PK model. AUC_ss_ values of the subjects in the placebo group were treated as zero (0). For efficacy (responder or non-responder) and safety (occurrence of gastrointestinal disorders or not) variables, relationships to PK parameters were examined using the logistic model as shown in Eq. (3) with SAS (Version 9.2, SAS Institute).

3 $$ \mathrm{Probability}\ \left(\mathrm{efficacy}\ \mathrm{or}\ \mathrm{safety}\ \mathrm{event}\right)=1/\left[1+\exp\ \left(-\mathrm{a}-\mathrm{b}\times \mathrm{PK}\ \mathrm{parameter}\right)\right] $$

where a and b are the parameters to be estimated.

## RESULTS

**Fig. 1:**
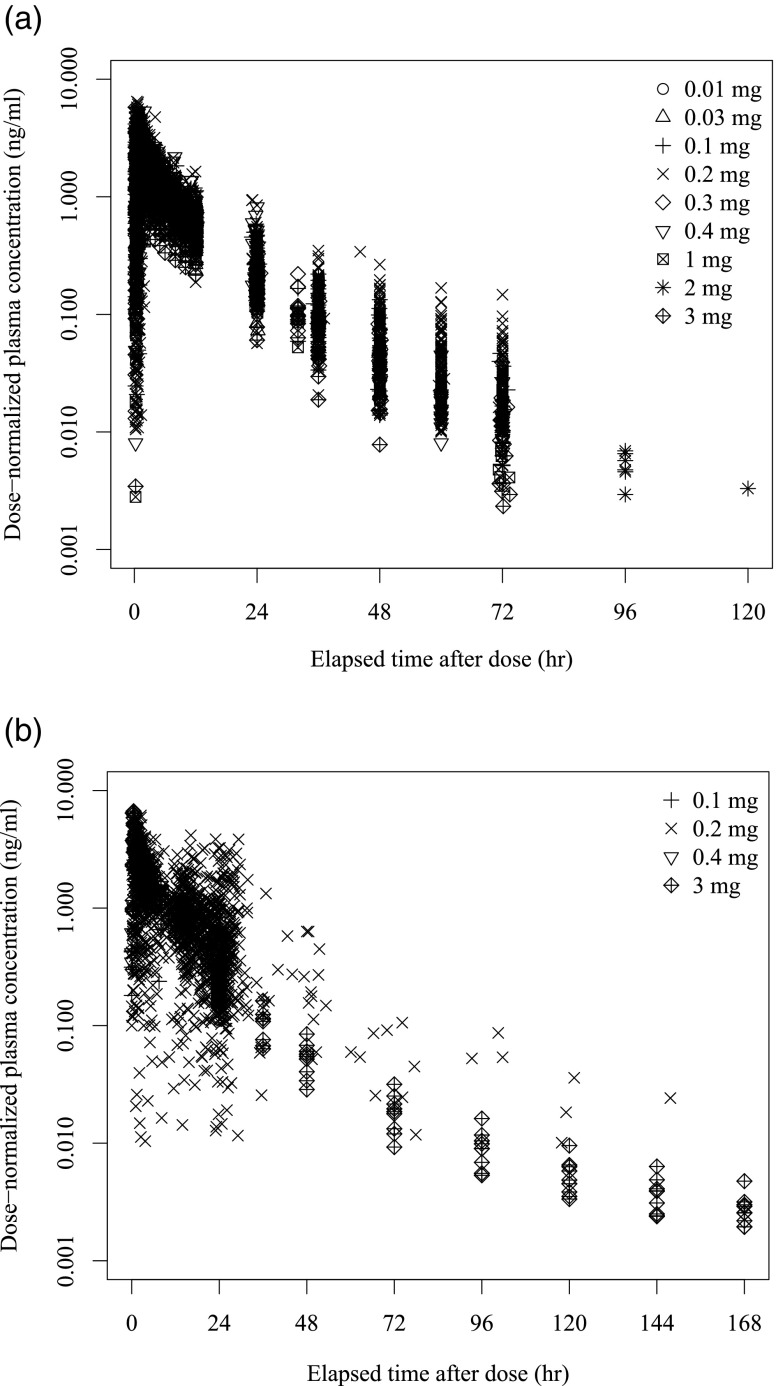
**Dose-normalized plasma naldemedine concentrations** ***versus*** **time after reference dose (dose-normalized to 0.2 mg)**. (**a**) Concentration profile after a single dose (**b**) Concentration profile after multiple doses.

A total of 9077 PK blood samples were obtained from the 1026 subjects. Of the 9077 plasma naldemedine concentrations, 39 data items were excluded for the following reasons;14 due to no measurement, 4 due to unidentified blood sampling time or unidentified dosing time, 2 due to detectable naldemedine concentration before the initial dose, and 19 due to their being unexpected outliers. Of the 9038 remaining concentrations, 892 below the limit of quantification (BLQ) records (402 from pre-dose and 490 from post-dose plasma samples) were treated as missing in the population PK analysis. Consequently, a total of 8146 naldemedine concentrations from 949 subjects were included in the analysis. Dose-normalized plasma naldemedine concentrations *versus* time are presented in Fig. 1. A summary of the observed concentrations included in the population PK analysis is provided in Table I. Table II shows the summary of background data.

**Table II:** Summary of Background Data

(a) Continuous covariates							
	N	Mean	SD	CV%	Max	Median	Min
Age (years)	949	51	14	28.1	90	52	18
Body weight (kg)	949	79.5	23.4	29.4	188.1	76.0	34.4
BMI (kg/m^2^)	949	28.1	7.2	25.5	58.8	26.9	14.4
ALBU (g/dL)	949	4.3	0.5	10.8	5.4	4.3	2.1
AST (U/L)	949	22	13	57.2	223	19	6
ALT (U/L)	949	22	15	69.7	212	18	2
Tbil (mg/dL)	949	0.5	0.3	55.0	2.4	0.4	0.04
CLcr (ml/min)	949	109.5	42.4	38.7	311.8	108.0	5.8
(b) Categorical covariates							
						N	%
Gender	Male					486	51.2
	Female					463	48.8
Race	American Indian or Alaska native					9	0.9
	Asian					251	26.4
	Black or African American					128	13.5
	Native Hawaiian or other Pacific islander					2	0.2
	White					558	58.8
	the Others					1	0.1
White	White					558	58.8
	non-White					391	41.2
Japanese	Japanese					249	26.2
	non-Japanese					700	73.8
Hispanic or Latino	Hispanic or Latino					96	10.1
	non-Hispanic or non-Latino					853	89.9
Food condition	Fasted					817	86.1
	Fed					132	13.9
P-gp inhibitor	without P-gp inhibitor					891	93.9
	with P-gp inhibitor					58	6.1
CYP3A inhibitor	without CYP3A inhibitor					892	94.0
	with strong CYP3A inhibitor					14	1.5
	with moderate CYP3A inhibitor					43	4.5
CYP3A inducer	without CYP3A inducer					933	98.3
	with strong CYP3A inducer					10	1.1
	with moderate CYP3A inducer					6	0.6
Formulation	Solution or suspension					54	5.7
	Phase 1 or 2 tablet					130	13.7
	Phase 3 tablet					765	80.6
Age	<65					783	82.5
	> = 65					166	17.5

*BMI* Body mass index, *ALBU* Albumin, *AST* Aspartate aminotransferase, *ALT* Alanine aminotransferase, *Tbil* Total bilirubin, *CLcr* Creatinine clearance

The model building process is provided in Supplemental Table S2. A two-compartment model with first-order absorption was selected as a structural PK model because the naldemedine concentration appeared to decline in a biphasic manner, and the pharmacokinetic parameters could not be stably estimated with a three-compartment model in the NONMEM calculation. An absorption lag time (ALAG) was incorporated into the model based on the OBJ value. The structural PK parameters were CL/F, Vc/F, Q/F, Vp/F, Ka, and ALAG. The inter-individual variability for ALAG was removed from the model to allow convergence of the estimation and covariance routines. Based on the OBJ, the proportional error model was chosen for intra-individual variability. The parameter estimates of the base model are given in Supplemental Table S3. Supplemental Fig. S1 shows the goodness-of-fit plots for the base model.

**Fig. 2:**
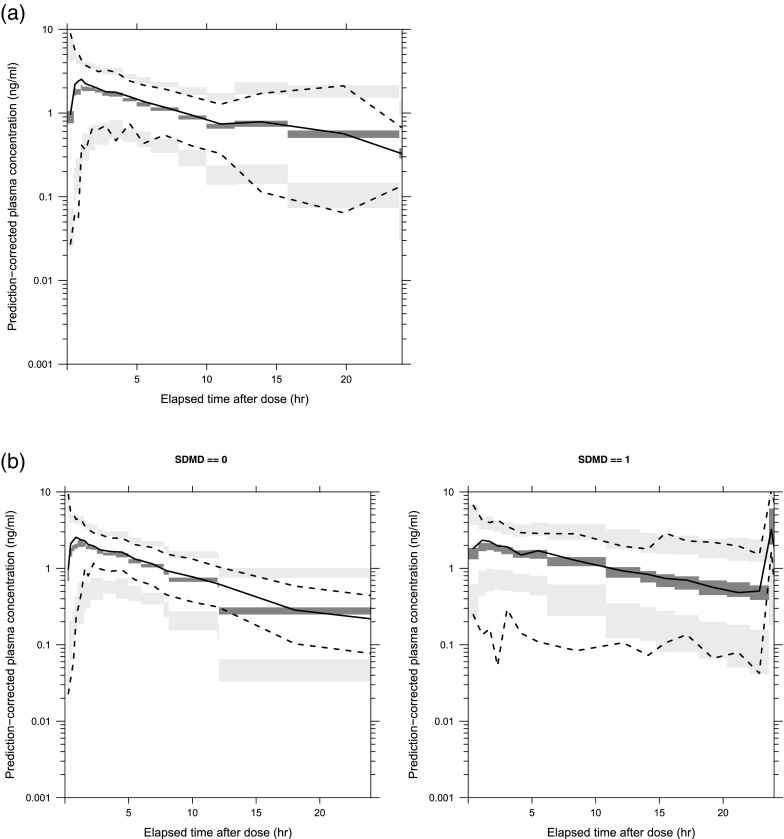
**Visual predictive check for the final model**. (Solid line: observed median. Dotted line: observed 2.5th and 97.5th percentiles. Dark grey shaded area: model predicted 95% confidence interval of median. Gray shaded area:model predicted 95% confidence intervals of 2.5th and 97.5th percentiles.). (**a**) All (**b**) Stratified by Single and Multiple Doses (Left: Single dose, Right: Multiple doses).

The effects of the covariates on principal PK parameters (CL/F, Vc/F, and Ka) were investigated using the forward selection and stepwise backward elimination procedure (Supplemental Table S2). The final model included the effects of age, CLcr, race (White or non-White), and gender on CL/F; the effects of body weight, health status, and food condition on Vc/F; and the effect of age on Ka. The parameter estimates of the final model are presented in Table III together with the bootstrap estimates and confidence intervals. The population parameter estimates were: CL/F = 9.10 L/h, Vc/F = 91.1 L, Ka = 2.94 h^−1^, Q/F = 4.77 L/h, Vp/F = 41.8 L, and ALAG = 0.195 h for the reference population; which was 52-year-old, 76 kg male, white, non-cancer, OIC patients with CLcr of 108 ml/min, administed under fasted condintion. The typical AUC_ss_ value for the reference population was 21.98 ng·hr/ml with the regimen of naldemedine 0.2 mg once daily. A summary of the empirical Bayesian-estimated CL/F and AUC_ss_ is presented in Supplemental Table S4. Supplemental Fig. S2 shows the goodness-of-fit plots for the final model. Figure 2 presents the results of the VPC for the final model in the time range from 0 to 24 h post-dose. The concentration profiles of the observed median, 2.5th, and 97.5th percentiles for the naldemedine concentrations were well captured by the 95% prediction interval of the corresponding estimated percentiles, respectively, and the calculated percentage of the observations outside the 90% prediction interval was 7.4%. The relationships between the inter-individual variabilities on each PK parameter were provided in Supplemental Fig. S3. There was no clear correlation between the inter-individual variabilities.

**Table III:** Population PK Parameter Estimates for the Final Model

	Estimate	Shrinkage	95% confidence interval			Median and 95% confidence interval for bootstrap estimates			
Pharmacokinetic model			Lower		Upper	Median	Lower		Upper
CL/F (L/h)	CL/F = THETA (1) * (Age/52) ** THETA (2) * (CLcr/108) ** THETA (3) * THETA (4) ** White * THETA (5) ** Gender								
THETA (1)	9.10		8.73	–	9.47	9.15	8.77	–	9.77
THETA (2)	−0.195		−0.291	–	−0.0986	−0.189	−0.256	–	−0.103
THETA (3)	0.0739		−0.0133	–	0.161	0.0781	−0.00460	–	0.165
THETA (4)	0.870		0.820	–	0.920	0.872	0.807	–	0.920
THETA (5)	0.902		0.857	–	0.947	0.895	0.856	–	0.961
Vc/F (L)	Vc/F = THETA (6) * (Body weight/76) * THETA (7) ** non-Cancer * THETA (8) ** Cancer * THETA (9) ** Food condition								
THETA (6)	75.9		73.2	–	78.6	76.0	72.7	–	78.4
THETA (7)	1.20		1.12	–	1.28	1.19	1.11	–	1.26
THETA (8)	1.27		1.05	–	1.49	1.28	1.15	–	1.38
THETA (9)	1.12		1.05	–	1.19	1.12	1.05	–	1.22
Ka (hr^−1^)	Ka = THETA (10) * (Age/52) ** THETA (11)								
THETA (10)	2.94		2.32	–	3.56	2.90	2.43	–	3.51
THETA (11)	−1.16		−1.26	–	−1.06	−1.23	−1.49	–	−1.10
Q/F (L/h)	4.77		4.16	–	5.38	4.73	4.16	–	5.29
Vp/F	41.8		38.4	–	45.2	41.6	38.5	–	44.3
ALAG (hr)	0.195		0.188	–	0.202	0.196	0.190	–	0.198
Inter-individual variability (CV%)									
CL/F	37.9	6.6	35.2	–	40.5	38.6	34.9	–	41.6
Vc/F	25.3	40.5	20.8	–	29.2	25.2	22.4	–	28.5
Ka	161.2	32.6	142.7	–	177.9	161.0	145.1	–	176.0
Q/F	46.3	60.6	29.9	–	58.2	47.5	30.0	–	64.8
Vp/F	36.3	57.1	30.2	–	41.6	36.2	30.6	–	42.0
Intra-individual variability (CV%)									
proportional	25.7	10.8	24.5	–	26.9	25.5	24.4	–	26.7

*CL/F* Apparent total clearance, *Vc/F* Apparent volume of central compartment, *Q/F* Apparent inter-compartmental clearance, *Vp/F* Apparent volume of peripheral compartment, *Ka* First-order rate of absorption, *ALAG* Absorption lag time

White = 1 for non-White, White = 0 for White; Gender = 1 for female, Gender = 0 for male; non-Cancer = 1 and Cancer = 0 for patients with chronic non-cancer pain and OIC, non-Cancer = 0 and Cancer = 1 for cancer patients with OIC, non-Cancer = 0 and Cancer = 0 for healthy subjects; Food = 1 for fed condition, Food = 0 for fasted condition

The frequency of the occurrence of SBM responders for the PK/Efficacy analysis and the frequency of gastrointestinal disorders in the PK/Safety analysis are provided in Table IV. The logistic model was used for the PK/Efficacy analysis and the PK/Safety analysis. The parameter estimates of logistic analysis are shown in Table V.

**Table IV:** Frequency of Spontaneous Bowel Movement Responders and Gastrointestinal Disorders

Spontaneous bowel movement responder in the PK/efficacy population^a^					
Study No.	Dose (mg)		Non_Responder		Responder
1107 V9221 (Phase 2b)	0		37 (60.7)		24 (39.3)
	0.1		4 (44.4)		5 (55.6)
	0.2		3 (33.3)		6 (66.7)
	0.4		4 (40.0)		6 (60.0)
1314 V9231 and 1315 V9232 (Phase 3)	0		361 (65.9)		187 (34.1)
	0.2		208 (46.7)		237 (53.3)
Gastrointestinal disorders in the PK/safety population^b^					
Study No.	Dose (mg)	Not_Reported	Mild	Moderate	Severe
1107 V9221 (Phase 2b)	0	53 (86.9)	6 (9.8)	2 (3.3)	0 (0.0)
	0.1	7 (77.8)	1 (11.1)	1 (11.1)	0 (0.0)
	0.2	5 (55.6)	1 (11.1)	3 (33.3)	0 (0.0)
	0.4	7 (70.0)	2 (20.0)	1 (10.0)	0 (0.0)
1314 V9231 and 1315 V9232 (Phase 3)	0	473 (86.3)	47 (8.6)	21 (3.8)	7 (1.3)
	0.2	351 (78.9)	52 (11.7)	34 (7.6)	8 (1.8)

^a^Count (Percent)

^b^Count (Row percent)

**Table V:** Parameter Estimates of PK/Efficacy Analysis and PK/Safety Analysis

(a) PK/efficacy analysis for spontaneous bowel movement (SBM) responder					
Study No.	Parameter	Estimate		95% confidence interval	
				Lower	Upper
1107 V9221 (Phase 2b)	a	−0.314		−0.797	0.159
	b	0.0191		−0.00801	0.0497
1314 V9231 and 1315 V9232 (Phase 3)	a	−0.537		−0.700	−0.375
	b	0.0194		0.0114	0.0274
(b) PK/safety analysis for gastrointestinal disorders					
Study No.	Severity of Gastrointestinal Disorders	Parameter	Estimate	95% Confidence Interval	
				Lower	Upper
1107 V9221 (Phase 2b)	Mild, Moderate, Severe	a	−1.76	−2.46	−1.16
		b	0.0310	0.00138	0.0617
	Moderate, Severe	a	−2.75	−3.84	−1.90
		b	0.0254	−0.0170	0.0615
1314 V9231 and 1315 V9232 (Phase 3)	Mild, Moderate, Severe	a	−1.72	−1.95	−1.51
		b	0.0106	0.000712	0.0202
	Moderate, Severe	a	−2.75	−3.09	−2.44
		b	0.0125	−0.00169	0.0259
	Severe	a	−4.39	−5.15	−3.77
		b	0.015	−0.0152	0.0409

^a^Probability (SBM Responder) = 1 / [1 + exp. (− a - b × AUC_ss_)]

^b^Probability (Gastrointestinal Disorders) = 1 / [1 + exp. (− a - b × AUC_ss_)]

Table VI provides the probabilities of the SBM responder and the occurrence of gastrointestinal disorders for the placebo and the doses of 0.1, 0.2, and 0.4 mg calculated with the mean AUC_ss_ based on the developed logistic model. When subjects took the placebo or 0.2 mg of naldemedine, the probabilities of SBM responders were predicted to be 42.2% for the placebo and 52.7% for the 0.2 mg dose in the phase 2b study (Study No. 1107 V9221); and 36.9% for the placebo and 49.9% for the 0.2 mg dose in the phase 3 studies (Study No. 1314 V9231 and 1315 V9232). In the phase 3 study, the probabilities of the occurrence of gastrointestinal disorders with the placebo or the 0.2 mg dose were predicted to be 15.1 and 19.3% for mild to severe gastrointestinal disorders; 6.0 and 8.3% for moderate to severe gastrointestinal disorders; and 1.2 and 1.8% for severe gastrointestinal disorders, respectively. In the phase 2b study, the calculated probabilities were similar to those in the phase 3 studies although the probability of the occurrence of severe gastrointestinal disorders was not estimated because none were reported. These probabilities were comparable with the corresponding observed frequency.

**Table VI:** Probabilities Predicted from PK/Efficacy Models and PK/Safety Models

Probabilities of occurrence of spontaneous bowel movement (SBM) responder^a^										
Study No.	Placebo		Dose: 0.1 mg				Dose: 0.2 mg		Dose: 0.4 mg	
	AUC_ss_	Probability	AUC_ss_		Probability		AUC_ss_	Probability	AUC_ss_	Probability
1107 V9221 (Phase 2b)	0.00	0.422	11.06		0.474		22.11	0.527	44.22	0.630
1314 V9231 and 1315 V9232 (Phase 3)	0.00	0.369	13.75		0.433		27.50	0.499	55.00	0.630
Probabilities of occurrence of gastrointestinal disorders^b^										
Study No.	Severity of Gastrointestinal Disorders		Placebo		Dose: 0.1 mg		Dose: 0.2 mg		Dose: 0.4 mg	
			AUC_ss_	Probability	AUC_ss_	Probability	AUC_ss_	Probability	AUC_ss_	Probability
1107 V9221 (Phase 2b)	Mild, Moderate, Severe		0.00	0.146	11.06	0.195	22.11	0.254	44.22	0.403
	Moderate, Severe		0.00	0.060	11.06	0.078	22.11	0.101	44.22	0.165
1314 V9231 and 1315 V9232 (Phase 3)	Mild, Moderate, Severe		0.00	0.151	13.75	0.172	27.50	0.193	55.00	0.242
	Moderate, Severe		0.00	0.060	13.75	0.071	27.50	0.083	55.00	0.113
	Severe		0.00	0.012	13.75	0.015	27.50	0.018	55.00	0.027

^a^Probability (SBM Responder) = 1 / [1 + exp. (− a - b × AUC_ss_)]

^b^Probability (Gastrointestinal Disorders) = 1 / [1 + exp. (− a - b × AUC_ss_)]

AUC_ss_ in the placebo group was treated as zero. AUC_ss_ in 0.1 mg group was assumed to be half the AUC_ss_ in 0.2 mg group

AUC_ss_ in 0.4 mg group was assumed to be double the AUC_ss_ in 0.2 mg group

Unit of AUC_ss_: ng*hr/ml

AUC in the placebo group was treated as zero. AUC in 0.1 mg group was assumed to be half the AUC in 0.2 mg group

AUC in 0.4 mg group was assumed to be double the AUC in 0.2 mg group

Unit of AUC: ng*hr/ml

The clinical relevance of the covariates in the population model could be evaluated by the exposure-response model. If the covariates independently took extreme values within the ranges or categories of age (18–90 years), CLcr (5.8–311 ml/min), race (White, non-White), and gender (male, female) in the analysis population, the population predicted CL/F would range from 5.17 to 12.1 L/h, which would decrease the population predicted AUC_ss_ by 25% or would increase by 76% compared with that of the reference population. These alterations in PK would not be considered clinically significant because similar efficacy and safety would be expected for 1/2-fold or 2-fold alteration of exposure based on the exposure-response analysis. In addition, Supplemental Table S5 provides the summary of the empirical Bayesian-estimated CL/F in the pivotal phase 3 studies (Study No. 1314 V9231 and 1315 V9232) by categorical age (<65, ≥65 years or < 65, 65- < 75, ≥75 years), gender, race, or health status. There was no clear difference in CL/F among categorical age, gender, race, and health status. Consequently, no dose-adjustment based on the covariates is necessary.

## DISCUSSION AND CONCLUSIONS

This study characterizes the population PK of naldemedine with data from phase 1 through 3 studies. The PK/Efficacy and PK/Safety models were built with data from phase 2b and 3 studies. The population PK model provides information about the influential factors on naldemedine PK. The PK/Efficacy and PK/Safety models describe the exposure-response relationship for the clinical dose of 0.2 mg.

Naldemedine plasma concentrations were adequately fitted by the two-compartment model with first-order absorption and absorption lag time. The final population PK model adequately described the observations including the peak concentration, which could not be explained well by the base model. Based on the goodness-of-fit plots, observations tended to be higher than predicted for the concentrations at >100 h post-dose. Although the discrepancies suggested that the model could not describe those low concentration data well, they were not considered to be clinically meaningful because the concentrations were minute, being less than 1/100 of C_max_. Nonparametric bootstrap procedure with the final model was performed. Of 200 NONMEM runs, 98 runs (49.0%) were completed successfully. Bootstrap parameter estimates (median and 95% CI) are presented in Table III. The 95% CIs for bootstrap estimates were similar to those calculated from the standard errors. In the population PK analysis, 19 concentrations were excluded from the analysis dataset because they were unexpected outliers. The influence of this exclusion on the results was examined by sensitivity analysis in which these outliers were incorporated into the dataset with the data being analyzed with the final model. However, the NONMEM run was not completed successfully possibly because the outliers made the model less stable. The final model was evaluated by using the VPC, and the observed concentration profiles were well captured by the final model. They supported the conclusion that the final model adequately described the observed data.

The final model included the effect of age, CLcr, race (White or non-White), and gender on CL/F; the effect of body weight, health status, and food condition on Vc/F; and the effect of age on Ka. The correlations between the covariates are given in Supplemental Fig. S4 and Supplemental Table S6. There was a weak correlation between age and CLcr, and the 95% CI of the estimate of CLcr on CL/F included 0, but it was not excluded from the final model because a difference in the AUC for naldemedine between healthy subjects with normal renal function and subjects with renal impairment was observed in a renal impairment study (8). The relationships between continuous variables and categorical variables were evaluated by analysis of variance. Statistically significant relationships (*p* < 0.05) were observed between variable pairs except for age and race (White or non-White) probably because a large number of subjects (*n* = 949) were included in the population pharmacokinetic analysis. There was no trend for those covariate pairs in Supplemental Fig. S4. Therefore, the covariates were incorporated in the model based on the value of OBJ.

Efficacy and safety related to the alteration in exposure due to the covariates in the population PK analysis can be evaluated based on the exposure-response model. In the PK/Efficacy and PK/safety analysis, the data from the phase 2b study and the phase 3 studies were separately analyzed since the study duration and definition of SBM responders were different. In this paper, the exposure-response models were built with AUC_ss_. The AUC_ss_ and C_max_ were highly correlated (Supplemental Fig. S5) and similar results were obtained with C_max_ (data not shown). The developed models can predict the probability of there being SBM responders and the occurrence of gastrointestinal disorder based on AUC_ss_ as illustrated in Supplemental Fig. S6. The probability of SBM responders was comparable between the phase 2b and phase 3 studies. On the other hand, the probabilities of the occurrence of gastrointestinal disorders in the phase 2b study were different from those in the phase 3 study because the model parameters from the phase 2b study could not be estimated properly due to the small sample size. The probability of the occurrence of severe gastrointestinal disorders with a naldemedine dose of 0.2 mg and 0.4 mg were predicted as being less than 3% in the phase 3 study. These results suggest that there is a weak positive correlation between the probability of SBM responders and naldemedine AUC_ss_ and that minimal differences in the proportion of SBM responders could be predicted for 1/2-fold or 2-fold mean AUC_ss_ at a dose of 0.2 mg.

Some limitations exist in this study. First, covariate model was constructed by means of combination of forward selection after screening and stepwise backward deletion. Since the forward selection procedure tested the covariates in order of magnitude of the decrease in OBJ in screening, some combinations of covariate effects were not evaluated. The other limitation is that the 95% CI of the slope contained 0 in the exposure-response analysis. In the PK/Efficacy analysis, the lower limit of the 95% CI of the slope was slightly lower than 0 (−0.00801) for the phase 2b study. It implied the estimated value for the slope was not statistically different from 0 and the probability might be independent of AUC_ss_. This is probably due to the insufficient suboptimal data for the logistic regression since the high proportion of the SBM responder was observed at the lowest naldemedine dose of 0.1 mg. In addition, the 95% CI of the slope also contained 0 in PK/safety analysis in Table V although dose-dependent increases in gastrointestinal-related adverse reactions had been reported in a previous study in which single dose of naldemedine up to 3 mg (15 times the recommended dose) had been administered to patients with OIC (8). This is probably because the frequency of the moderate or severe adverse event was insufficient to develop those models. Thus, the slopes appeared to be flat since there were no issues in the exposure range from the dose from 0.1 to 0.4 mg which is 1/2-fold or 2-fold of clinical dose of 0.2 mg. Moreover, the probabilities predicted from the model were comparable with the observations as summarized in Supplemental Table S7. Therefore, we concluded the exposure-response models can be used to interpret the exposure response relationship in the phase 2b study (Study No. 1107 V9221) and the phase 3 studies (Study No. 1314 V9231 and 1315 V9232); however it is inappropriate to generalize them because the data were obtained in the limited exposure range.

In conclusion, naldemedine plasma concentrations can be adequately described by the 2-compartment model with first-order absorption and absorption lag time. The age, CLcr, race (White or non-White), gender, body weight, health status, and food condition influence naldemedine PK. The PK/Efficacy and the PK/Safety models were developed using logistic regression. When subjects took 0.2 mg of naldemedine in the phase 2b and phase 3 studies, the probability of SBM responders was predicted to be approximately 50%, and the probability of the occurrence of severe gastrointestinal disorders was predicted to be less than 3%. Similar probabilities were calculated at naldemedine doses of 0.1 mg and 0.4 mg. The exposure-response analysis suggests minimal differences in the proportion of SBM responders and severe gastrointestinal disorders for 1/2-fold or 2-fold AUC_ss_ at 0.2 mg. Therefore, no dose-adjustment is required for the selected covariates based on the exposure-response analysis.

## Electronic supplementary material

ESM 1 (DOCX 592 kb)

ESM 2 (DOCX 113 kb)

## ABBREVIATIONS

ALAG: Absorption lag time
ALBU: Albumin
ALT: Alanine aminotransferase
AST: Aspartate aminotransferase
AUC: Area under the plasma concentration curve
AUC_ss_: Area under the plasma concentration curve at steady-state
BLQ: Below the limit of quantification
BMI: Body mass index
CL/F: Apparent total clearance
CLcr: Creatinine clearance
C_max_: Maximum plasma concentration
Ka: First-order rate of absorption
OBJ: Objective function value
OIC: Opioid-induced constipation
PK: Pharmacokinetics
Q/F: Apparent inter-compartmental clearance
SBM: Spontaneous bowel movement
Tbil: Total bilirubin
V/F: Apparent volume of central compartment
Vp/F: Apparent volume of peripheral compartment
VPC: Prediction-corrected visual predictive checks
