# Supplementary material for: Population Pharmacokinetics and Exposure-Response Relationships of Naldemedine
Source: Pharm Res. 2018 Oct 2;35(11):225. doi: 10.1007/s11095-018-2501-7 (PMC6182381; doi:10.1007/s11095-018-2501-7)

Supplemental Figure S1 Goodness-of-Fit Plots for the Base Model


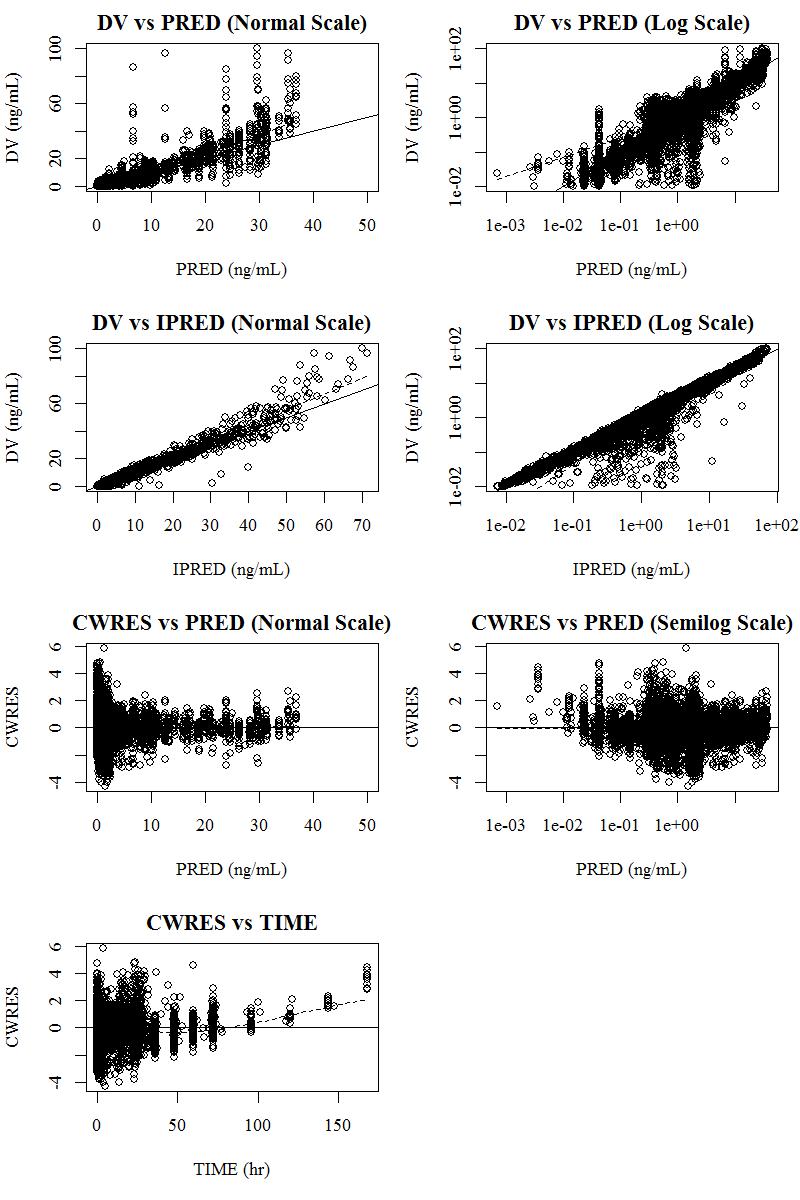


DV = Observed concentrations

PRD = Population predicted concentrations

IPRED = Bayesian-predicted individual concentrations

CWRES = Conditional weighted residuals

Supplemental Figure S2 Goodness-of-Fit Plots for the Final Model


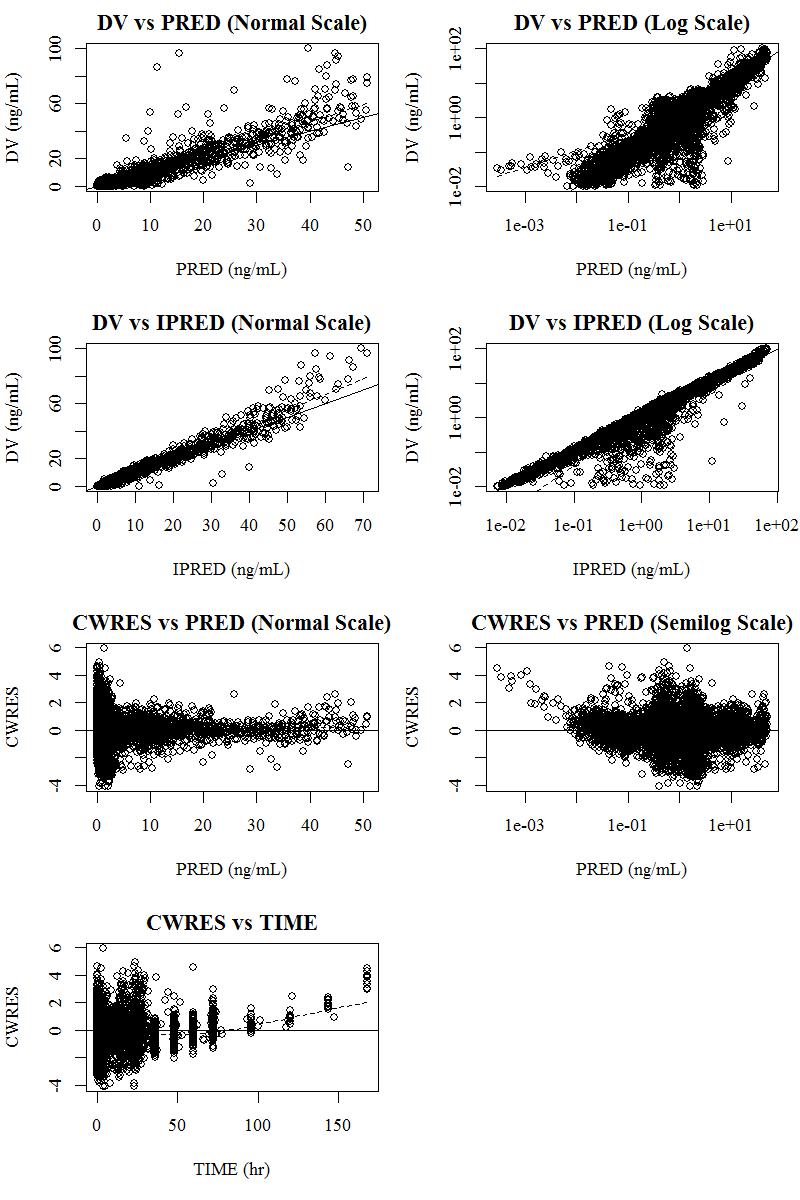


DV = Observed concentrations

PRD = Population predicted concentrations

IPRED = Bayesian-predicted individual concentrations

CWRES = Conditional weighted residuals

Supplemental Figure S3 Plots and Coefficient of Correlation between the inter-individual variabilities on each PK parameter in the final model


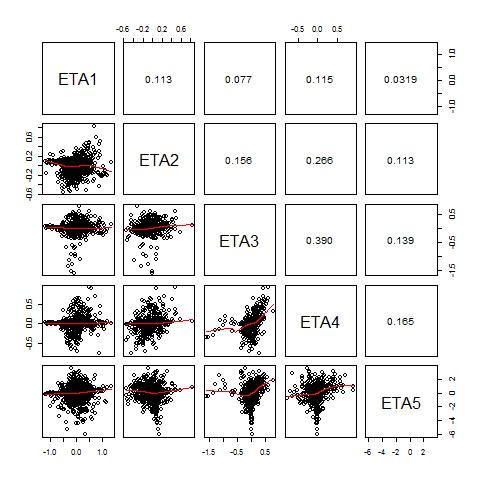


Supplemental Figure S4 Plots of the Covariate Pairs in the Final Population PK Model


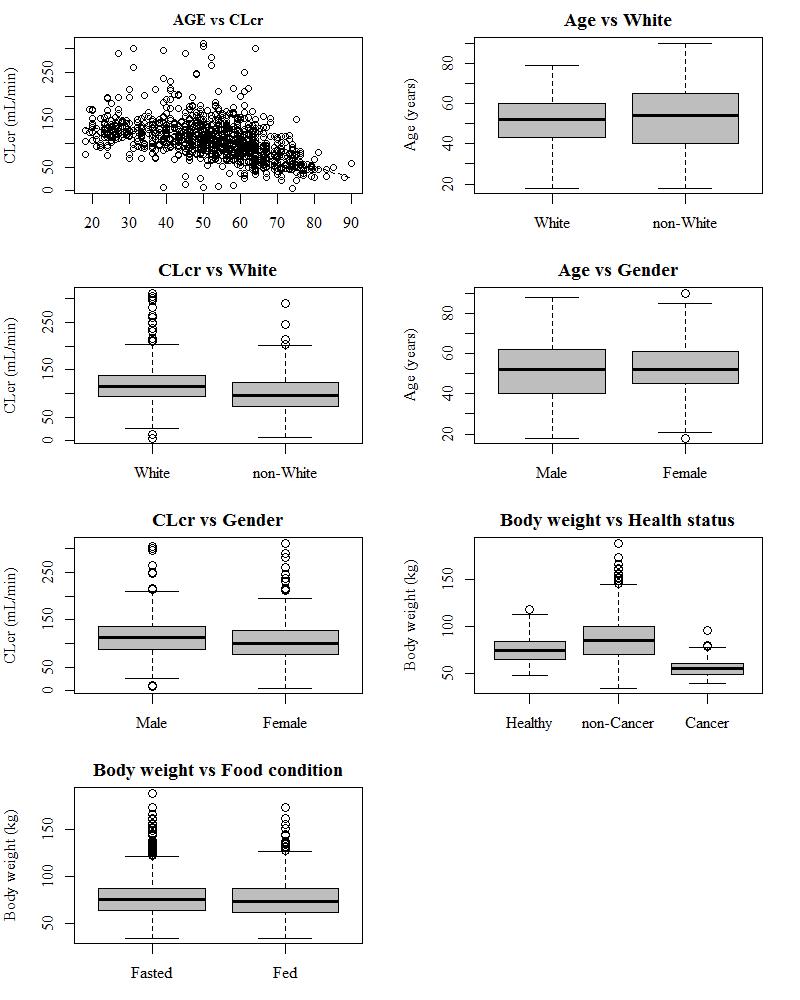


Supplemental Figure S5 Plots of Empirical Bayesian-Estimated AUC_ss_ and C_max_

1. Phase 2b study (1107V9221)


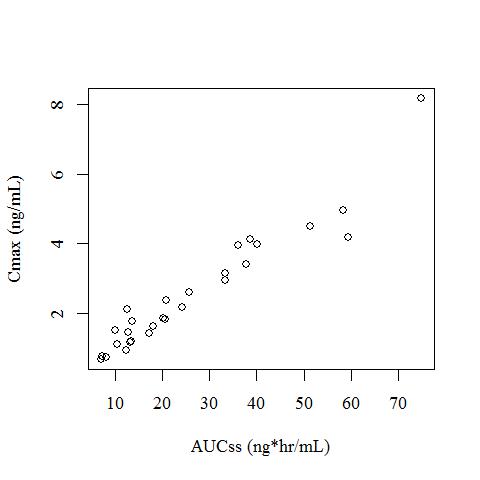


(b) Phase 3 studies (1314V9231 and 1315V9232)


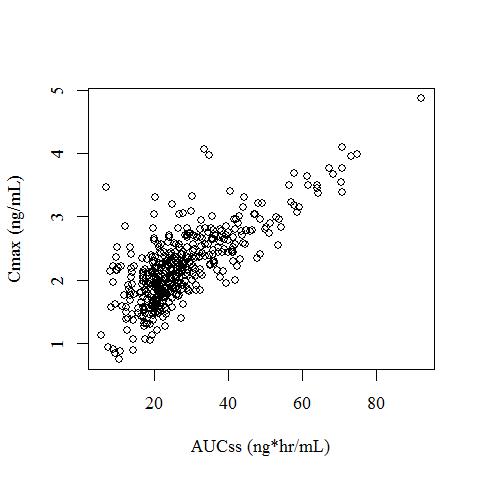


Supplemental Figure S6 Plots of Logistic Regression for Spontaneous Bowel Movement (SBM) Responders and Gastrointestinal (GI) Disorders

(Solid Line: SBM Responder. Dotted Line: Mild, Moderate, and Severe GI Disorders. Dot-Dashed Line: Moderate and Severe GI Disorders. Dashed Line: Severe GI Disorders. Blue vertical lines: Mean AUC_ss_ at each Dose)

(a) Phase 2b Study with Efficacy Observation (Open Circle: Responder. Cross: Non-Responder)


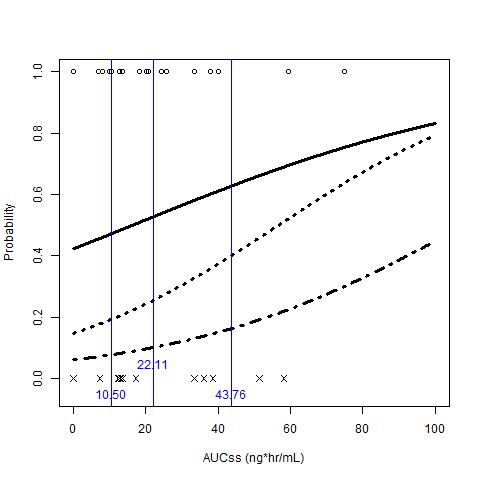


(b) Phase 2b Study with Severity of Gastrointestinal Disorders (Open Circle: Not_Reported. Square: Mild. Triangle: Moderate)


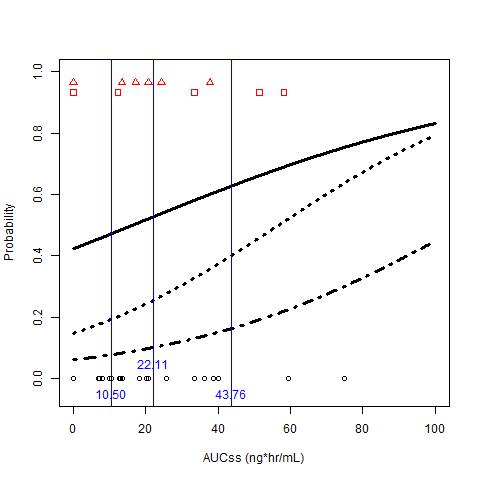


Supplemental Figure S6 (Continued)

(c) Phase 3 Studies with Efficacy Observation (Open Circle: Responder. Cross: Non-Responder.)


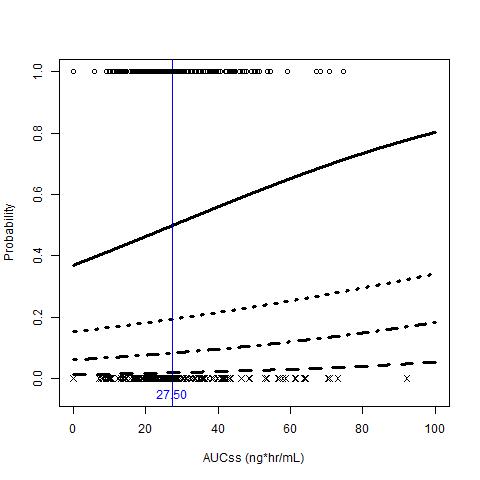


(d) Phase 3 Studies with Severity of Gastrointestinal Disorders (Open Circle: Not_Reported. Square: Mild. Triangle: Moderate. Cross: Severe.)


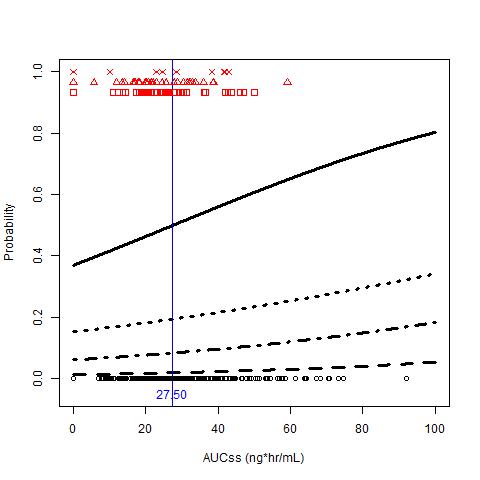

Supplement: Supplementary file 1 — (DOCX 592 kb) [file 11095_2018_2501_MOESM1_ESM.docx]
