# Supplementary material for: Population Pharmacokinetics and Exposure-Response Relationships of Naldemedine
Source: Pharm Res. 2018 Oct 2;35(11):225. doi: 10.1007/s11095-018-2501-7 (PMC6182381; doi:10.1007/s11095-018-2501-7)
Supplement: Supplementary file 2 — (DOCX 113 kb) [file 11095_2018_2501_MOESM2_ESM.docx]

Supplemental Table S1 Blood Sampling Time for Pharmacokinetic Analysis

| **Study Title (Study No.)** | **Blood Sampling Time for Pharmacokinetic Analysis** |
| --- | --- |
| Single Dose Study (Healthy Japanese) (0824V9211) | 0, 0.25, 0.5, 1, 1.5, 2, 2.5, 3, 3.5, 4, 4.5, 5, 6, 8, 10, 12, 24 and 32 hours post-dose |
| Multiple Dose Study (Healthy Japanese) (0917V9213) | Days 1 and 10: 0, 0.25, 0.5, 0.75, 1, 1.5, 2, 2.5, 3, 4, 5, 6, 8, 10 and 12 hours  Days 2 to 9: 0 hours  Days 11 to 17: 24, 36, 48, 72, 96, 120, 144 and 168 hours post dose |
| Mass Balance Study (1016V9215) | 0, 0.25, 0.5, 0.75, 1, 1.5, 2, 2.5, 3, 4, 5, 6, 8, 10, 12, 24, 36, 48, 72, 96 and 120 hours. |
| DDI Study with Cyclosporine (P-gp Inhibitor) (1202V9218) | 0, 0.25, 0.5, 0.75, 1, 1.5, 2, 2.5, 3, 4, 5, 6, 8, 10, 12, 24, 36, 48, 60 and 72 hours post-dose |
| Thorough QTc Study (1204V9219) | 0, 0.25, 0.5, 1, 1.5, 2, 3, 4, 6, 12 and 24 hours post-dose |
| BA/ FE Study (To-be-marketed Tablet) (1311V921A) | 0, 0.25, 0.5, 0.75, 1, 1.5, 2, 2.5, 3, 4, 5, 6, 8, 10, 12, 24, 36, 48, 60 and 72 hours  post-dose |
| DDI Study with Rifampin (CYP3A Inducer) (1403V921D) | 0, 0.25, 0.5, 0.75, 1, 1.5, 2, 2.5, 3, 4, 5, 6, 8, 10, 12, 24, 36, 48, 60 and 72 hours post-dose |
| Renal Impairment Study (1401V921B) | 0, 0.25, 0.5, 0.75, 1, 1.5, 2, 2.5, 3, 4, 5, 6, 8, 10, 12, 24, 36, 48, 60 and 72 hours post-dose |
| Hepatic Impairment Study (1402V921C) | 0, 0.25, 0.5, 0.75, 1, 1.5, 2, 2.5, 3, 4, 5, 6, 8, 10, 12, 24, 36, 48, 60 and 72 hours post-dose |
| DDI Study with Itraconazole/ Fluconazole (CYP3A Inhibitors) (Healthy Japanese) (1502V921E) | 0, 0.25, 0.5, 0.75, 1, 1.5, 2, 2.5, 3, 4, 5, 6, 8, 10, 12, 24, 36, 48, 60 and 72 hours post-dose |
| Phase 2 OBD POC Study in Patients with Chronic Non-cancer Pain (1007V9214) | 0, 0.25, 0.5, 0.75, 1, 1.5, 2, 2.5, 3, 4, 5, 6, 8, 10, 12, 24, 36, 48 and 72 hours post-dose |
| Phase 2b Dose Finding Study in Patients with Chronic Non-cancer Pain (1107V9221) | Day 1: 0, 1, 2, 4, 8 and 24 hours post-dose  Day 28: 0, 1, 2, 4, 8 and 24 hours post-dose |
| Phase 2b Dose Finding Study in Cancer Patients (1108V9222) | Day 1: 1, 2, 4, 8, 12 and 24 hours post-dose |
| Phase 3 DBT Study #1in Patients with Chronic Non-cancer Pain (1314V9231) | One point at Weeks 4, 8 and 12 |
| Phase 3 DBT Study #2 in Patients with Chronic Non-cancer Pain (1315V9232) | One point at Weeks 4, 8 and 12 |
| Phase 3 DBT Study in Japanese Cancer Patients (1331V9236) | Post dose (0.5 - 4 hours) on Day 1 and one point at Weeks 1 and 2 |
| Phase 3 Long-term Safety Study in Japanese Patients with Chronic Non-cancer Pain (1336V9238) | Post dose (0.5 - 4 hours) on Day 1 and one point at Week 2 |
| Phase 3 Long-term Safety Study, in Japanese Patients with Chronic Non-cancer Pain Receiving Oxycodone Therapy  (1339V9239) | Post dose (0.5 - 4 hours) on Day 1 and one point at Week 2 |
| Abbreviations: Bioavailability (BA); Double blind test (DBT); Drug-Drug Interaction (DDI); Food effect (FE); Opioid-induced bowel dysfunction (OBD); Opioid-induced constipation (OIC); Proof of concept (POC). | |

Supplemental Table S2 Model Building Process

(a) To Base Model

Supplemental Table S2 (Continued)

(b) Screening for Covariates - 1

Supplemental Table S2 (Continued)

(c) Screening for Covariates - 2

Supplemental Table S2 (Continued)

(d) Forward Selection - 1

Supplemental Table S2 (Continued)

(e) Forward Selection - 2

Supplemental Table S2 (Continued)

1. Backward Elimination - 1

Supplemental Table S2 (Continued)

1. Backward Elimination - 2

Supplemental Table S3 Population PK Parameter Estimates for the Base Model

|  | Estimate | Shrinkage | 95 % Confidence Interval | | |
| --- | --- | --- | --- | --- | --- |
| Pharmacokinetic model | |  | Lower |  | Upper |
| CL/F (L/hr) | 8.20 |  | 7.96 | - | 8.44 |
| Vc/F (L) | 83.6 |  | 79.7 | - | 87.5 |
| Ka (hr^-1^) | 3.81 |  | 3.17 | - | 4.45 |
| Q/F (L/hr) | 5.02 |  | 4.45 | - | 5.59 |
| Vp/F (L) | 43.2 |  | 39.8 | - | 46.6 |
| ALAG (hr) | 0.195 |  | 0.188 | - | 0.202 |
| Inter-individual variability (CV%) | | | |  |  |
| CL/F | 40.5 | 7.2 | 37.6 | - | 43.2 |
| Vc/F | 38.5 | 32.5 | 33.9 | - | 42.6 |
| Ka | 168.2 | 33.9 | 147.8 | - | 186.4 |
| Q/F | 50.5 | 58.9 | 33.0 | - | 63.3 |
| Vp/F | 37.7 | 56.7 | 31.1 | - | 43.3 |
| Intra-individual variability (CV%) | | | |  |  |
| proportional | 25.6 | 11.4 | 24.3 | - | 26.7 |
| Abbreviations: Apparent total clearance (CL/F); Apparent volume of central compartment (Vc/F); Apparent inter-compartmental clearance (Q/F); Apparent volume of peripheral compartment (Vp/F); First-order rate of absorption (Ka); Absorption lag time (ALAG). | | | | | |

Supplemental Table S4 Summary of Empirical Bayesian-Estimated AUC_ss_

| Study No. |  | 1107V9221  (Phase 2b) | | |  | 1314V9231_1315V9232  (Phase 3) |
| --- | --- | --- | --- | --- | --- | --- |
| Dose (mg) |  | 0.1 | 0.2 | 0.4 |  | 0.2 |
| N |  | 9 | 9 | 10 |  | 445 |
| Mean |  | 10.50 | 22.11 | 43.76 |  | 27.50 |
| SD |  | 2.569 | 11.56 | 15.27 |  | 12.53 |
| CV% |  | 24.5 | 52.3 | 34.9 |  | 45.6 |
| Max |  | 13.49 | 51.36 | 74.93 |  | 92.1 |
| Median |  | 10.38 | 20.25 | 38.18 |  | 24.47 |
| Min |  | 7.02 | 12.66 | 25.78 |  | 5.669 |
| Geometric Mean |  | 10.2 | 20.29 | 41.64 |  | 25.11 |
| CV% Geometric Mean |  | 26.3 | 42.4 | 33.3 |  | 44.5 |

Unit of AUC_ss_: ng*hr/mL

Supplemental Table S5 Summary of Empirical Bayesian-Estimated CL/F in Pivotal Phase 3 Studies (Study No. 1314V9231, 1315V9232, and 1331V9236) by Age, Gender, Race, or Health Status

|  |  |  |  | CL/F (L/hr) | | | | | | |
| --- | --- | --- | --- | --- | --- | --- | --- | --- | --- | --- |
|  |  |  |  | 1314V9231_1315V9232 ^a^ | | |  | 1331V9236 ^b^ | | |
| Category | | |  | N | Mean | SD |  | N | Mean | SD |
| Age | < 65 |  |  | 387 | 8.89 | 4.12 |  | 50 | 7.51 | 3.50 |
|  | 65 -< 75 |  |  | 47 | 7.63 | 3.56 |  | 38 | 7.15 | 2.15 |
|  | >= 75 |  |  | 11 | 7.54 | 2.29 |  | 9 | 7.16 | 2.61 |
|  | >= 65 |  |  | 58 | 7.61 | 3.34 |  | 47 | 7.15 | 2.21 |
| Gender | Male |  |  | 185 | 9.08 | 4.10 |  | 59 | 7.85 | 3.48 |
|  | Female |  |  | 260 | 8.48 | 4.00 |  | 38 | 6.54 | 1.53 |
| Race | White |  |  | 360 | 9.00 | 4.14 |  | 0 | NA | NA |
|  | non-White |  |  | 85 | 7.59 | 3.39 |  | 97 | 7.34 | 2.94 |
|  |  | Black |  | 79 | 7.51 | 3.16 |  | 0 | NA | NA |
|  |  | Asian |  | 2 | 13.1 | NA |  | 97 | 7.34 | 2.94 |
|  |  | Others |  | 4 | 6.36 | 3.29 |  | 0 | NA | NA |
| Health status | OIC non-Cancer |  |  | 445 | 8.73 | 4.05 |  | 0 | NA | NA |
|  | OIC Cancer |  |  | 0 | NA | NA |  | 97 | 7.34 | 2.94 |
| Abbreviations: Not Applicable (NA); Opioid-induced constipation (OIC).  a Patients with Chronic Non-cancer Pain (Phase 3)  b Cancer patients with pain (Phase 3) | | | | | | | | | | |

Supplemental Table S6 The correlations between the covariates

(a) The Results of Correlation Analysis between Continuous Variable Pairs

| **Continuous Variables** | **Continuous Variables** | **Coefficient of Correlation** |
| --- | --- | --- |
| Age | CLcr | -0.45338 |

(b) The Results of Analysis of Variance between Continuous Variable and Categorical Variable

| **Continuous Variables** | **Categorical Variables** | **P-value** |
| --- | --- | --- |
| Age | White / non-White | 0.3057 |
| CLcr | White / non-White | < 0.0001 |
| Age | Gender | 0.0166 |
| CLcr | Gender | 0.0008 |
| Body weight | Health status | < 0.0001 |
| Body weight | Food condition | 0.0005 |

Supplemental Table S7 The prediction from the PK/Efficacy and PK/Safety models and the corresponding observations

(a) Efficacy

| Study No. | Dose (mg) | Observation | Prediction |
| --- | --- | --- | --- |
| 1107V9221 (Phase 2b) | 0 | 39.3 | 42.2 |
|  | 0.1 | 55.6 | 47.4 |
|  | 0.2 | 66.7 | 52.7 |
|  | 0.4 | 60.0 | 63.0 |
| 1314V9231 and 1315V9232 (Phase 3) | 0 | 34.1 | 36.9 |
|  | 0.2 | 53.3 | 49.9 |

(b) Safety

| Study No. | Severity of  Gastrointestinal Disorders | Dose (mg) | Observation | Prediction |
| --- | --- | --- | --- | --- |
|  |  |  |  |  |
| 1107V9221 (Phase 2b) | Mild, Moderate, Severe | 0 | 13.1 | 14.6 |
|  |  | 0.1 | 22.2 | 19.5 |
|  |  | 0.2 | 44.4 | 25.4 |
|  |  | 0.4 | 30.0 | 40.3 |
|  | Moderate, Severe | 0 | 3.3 | 6.0 |
|  |  | 0.1 | 11.1 | 7.8 |
|  |  | 0.2 | 33.3 | 10.1 |
|  |  | 0.4 | 10.0 | 16.5 |
| 1314V9231 and 1315V9232 (Phase 3) | Mild, Moderate, Severe | 0 | 13.7 | 15.1 |
|  |  | 0.2 | 21.1 | 19.3 |
|  | Moderate, Severe | 0 | 5.1 | 6.0 |
|  |  | 0.2 | 9.4 | 8.3 |
|  | Severe | 0 | 1.3 | 1.2 |
|  |  | 0.2 | 1.8 | 1.8 |
